# Quality of life in pediatric acute myeloid leukemia: Report from the Children's Oncology Group

**DOI:** 10.1002/cam4.2337

**Published:** 2019-06-12

**Authors:** Rajaram Nagarajan, Robert Gerbing, Todd Alonzo, Donna L. Johnston, Richard Aplenc, Edward A. Kolb, Soheil Meshinchi, Lamia P. Barakat, Lillian Sung

**Affiliations:** ^1^ Division of Oncology Cincinnati Children's Hospital Medical Center Cincinnati Ohio; ^2^ The Children's Oncology Group Monrovia California; ^3^ Division of Biostatistics University of Southern California Los Angeles California; ^4^ Division of Oncology Children's Hospital of Eastern Ontario Ottawa Ontario; ^5^ Division of Oncology Children's Hospital of Philadelphia Philadelphia Pennsylvania; ^6^ Division of Oncology AI Dupont Wilmington Delaware; ^7^ Division of Oncology Seattle Children's Hospital Seattle Washington; ^8^ Division of Haematology/Oncology The Hospital for Sick Children Toronto Ontario

**Keywords:** acute myeloid leukemia, fatigue, patient‐reported outcome, pediatric, quality of life

## Abstract

**Introduction:**

Objectives were used to describe guardian proxy‐report and child self‐report quality of life (QoL) during chemotherapy for pediatric acute myeloid leukemia (AML) patients.

**Methods:**

Patients enrolled on the phase 3 AML trial AAML1031 who were 2‐18 years of age with English‐speaking guardians were eligible. Instruments used were the PedsQL Generic Core Scales, Acute Cancer Module, and Multidimensional Fatigue Scale. Assessments were obtained at the beginning of Induction 1 and following completion of cycles 2‐4. Potential predictors of QoL included the total number of nonhematological grade 3‐4 Common Terminology Criteria for Adverse Event (CTCAE) submissions.

**Results:**

There were 505 eligible guardians who consented to participate and 348 of their children provided at least one self‐report assessment. The number of submitted CTCAE toxicities was significantly associated with worse physical health summary scores (*β* ± standard error (SE) −3.00 ± 0.69; *P* < 0.001) and general fatigue (*β* ± SE −2.50 ± 0.66; *P* < 0.001). Older age was significantly associated with more fatigue (*β* ± SE −0.58 ± 0.25; *P* = 0.022). Gender, white race, Hispanic ethnicity, private insurance status, risk status, bortezomib assignment, and duration of neutropenia were not significantly associated with QoL.

**Discussion:**

The number of CTCAE toxicities was the primary factor influencing QoL among children with AML. Reducing toxicities should improve QoL; identifying approaches to ameliorate them should be a priority.

## 1 INTRODUCTION

Children receiving cancer treatment experience worse quality of life (QoL) compared to healthy children.^1^ Among pediatric cancer patients, those receiving more intensive chemotherapy may have worse QoL.^2^ Since pediatric acute myeloid leukemia (AML) patients receive very intensive chemotherapy resulting in prolonged episodes of neutropenia and frequent life‐threatening infection,^3^ their QoL may be particularly poor during the on‐therapy period. However, little is known about the serial assessment of QoL over multiple treatment phases in this context, and which aspects of QoL are most impaired. Understanding QoL in these children is important to identify if and when interventions are required to improve subjective health. This information may also facilitate treatment decision making when QoL is compared between treatment arms.

Consequently, we embedded an ancillary aim on the phase 3 Children's Oncology Group randomized trial AAML1031 designed to compare bortezomib and no bortezomib for children with newly diagnosed AML and to describe the safety of sorafenib for patients with FLT‐3 internal tandem duplication high allelic ratio (ITD HAR). We previously reported 2 methodological studies focused on compliance with, and challenges to reporting of QoL with this sub‐study.^4^, ^5^ In these reports, we identified that important reasons for noncompletion of QoL questionnaires included the patient being too ill, and passive or active refusal by the respondent. We also determined that using a centralized coordinator to enhance compliance was not effective at improving QoL questionnaire completion rates.

The primary objective was to describe guardian proxy‐report and child self‐report QoL at 4 time points during chemotherapy for pediatric AML patients. Secondary objectives were to identify factors associated with worse proxy‐report physical health, psychosocial health and fatigue, and to describe concordance between guardian and child QoL reports.

## 2 MATERIALS AND METHODS

### 2.1 Parent AML trial

AAML1031 was a Phase 3 Children's Oncology Group multi‐center trial for children with newly diagnosed AML that randomized patients to receive or not receive bortezomib and determined the safety of sorafenib in patients with FLT3 ITD HAR. The study included patients with de novo AML less than 30 years of age at enrollment.

Four cycles of intense chemotherapy were administered to patients with standard‐risk disease and consisted of Induction 1, Induction 2, Intensification 1, and Intensification 2. Those with high‐risk disease received 3 cycles of chemotherapy followed by best allogeneic donor hematopoietic stem cell transplantation (HSCT). For both standard and high‐risk disease, Induction 1 consisted of cytarabine 100 mg/m^2^/dose intravenous (IV) every 12 hours on days 1‐10; daunorubicin 50 mg/m^2^/dose IV on days 1,3 and 5; and etoposide 100 mg/m^2^/dose IV daily on days 1‐5 (ADE 10+3+5). For standard‐risk patients, Induction 2 consisted of the same chemotherapy as Induction I except that cytarabine was administered for 8 days (ADE 8+3+5). Intensification 1 was cytarabine 1 g/m^2^/dose IV every 12 hours on days 1‐5 and etoposide 150 mg/m^2^/dose IV daily on days 1‐5 (AE) while Intensification 2 was cytarabine 1 g/m^2^/dose IV every 12 hours on days 1‐4 and mitoxantrone 12 mg/m^2^/dose IV daily on days 3‐6 (MA). For patients with high‐risk disease, Induction 2 and Intensification 1 consisted of MA and AE respectively while Intensification 2 consisted of cytarabine 3 g/m^2^/dose IV every 12 hours on days 1,2 and 8,9 and *Escherichiae coli* L'asparaginase 6000 international units/m^2^/dose intramuscularly on days 2 and 9. This study was approved by the National Cancer Institute's Central Institutional Review Board (IRB) and all participating sites’ IRBs and was registered with Clinical Trials Registration as study #NCT00005881.^6^

### 2.2 QoL assessment

This aim assessed the QoL of children and adolescents treated with chemotherapy and HSCT for AML; this report focuses on assessments performed during chemotherapy only and not HSCT. Consent and assent to participate in the ancillary QoL aim were obtained at the same time as consent for the therapeutic study. Those eligible for the QoL aim were patients enrolled to AAML1031 who also met the following specific criteria: patient between 2 and 18 years of age at diagnosis and English‐speaking guardian. Participating guardians provided proxy assessments for all patients, while self‐report for patients ≥5 years of age who could understand English was optional. Thus, a subset of patients had both self‐report and proxy‐report scores. Participants were enrolled between June 2011 and May 2015.

The instruments used were the PedsQL 4.0 Generic Core Scales,^7^, ^8^ PedsQL 3.0 Acute Cancer Module^9^ and PedsQL Multidimensional Fatigue Scale^9^, ^10^; these scales measure generic QoL, cancer‐specific QoL and fatigue respectively. The 27‐item PedsQL 3.0 Acute Cancer Module assess the following 8 dimensions: pain and hurt, nausea, procedural anxiety, treatment anxiety, worry, cognitive problems, perceived physical appearance, and communication. Higher scores represent better QoL. They are reliable and valid in pediatric cancer patients.^7^, ^8^, ^9^, ^10^ For all instruments, the 1 month recall version was used. This analysis evaluated the following time points: (a) within 14 days of starting Induction 1; (b) ≥ day 21 of Induction 2 and before starting Intensification 1; (c) ≥ day 21 of Intensification 1 and before starting Intensification 2; and (d) 1 month (±7 days) from start of intensification 2. In other words, the time points were chosen such that the first assessment was as close to a baseline assessment as was feasible and for subsequent cycles, the recall period approximated time from chemotherapy initiation to neutrophil recovery and start of the next cycle.

Administration was in the outpatient clinic or inpatient setting and questionnaires were completed on paper. These were not completed at home or electronically. Reminders to complete questionnaires were provided to sites by way of delinquency reports. To supplement these, a patient‐reported outcome coordinator was implemented between August 2012 and August 2013.

Respondents that did not complete a questionnaire (guardian or child form) at a particular time point continued to participate in subsequent time points as long as consent to participate was not withdrawn. Children who turned 5 years of age during the study could provide self‐report assessments after this time point. Respondents stopped completing QoL assessments if they were removed from AAML1031 protocol therapy for any reason including induction failure, relapse or death, or if consent to participate was withdrawn.

### 2.3 Analytic plan

The QoL aim was closed to patient accrual on May 15, 2015 as the intended number of subjects had been enrolled. Data for analyses were frozen as of March 31, 2018. Patients with FLT‐3 ITD HAR were eligible for the Phase I sorafenib treatment arm, and as this treatment arm remains under the purview of the Data Safety Monitoring Committee, data cannot be reported at this time. Demographic characteristics at study enrollment were summarized for patients who had submitted at least one guardian proxy‐report assessment or one child self‐report assessment at any of the 4 time points. Median and interquartile range were calculated for each assessment.

In order to evaluate factors associated with worse QoL, repeated measures linear regression with an unstructured covariance and random intercept was used. We modelled the specified proxy assessment scores to a potential predictor, time point, and the interaction between the potential predictor and time point. Scores of interest specified a priori were physical health summary score and psychosocial health summary score from the PedsQL 4.0 Generic Core Scales, and general fatigue scores from the PedsQL Multidimensional Fatigue Scale. The physical and psychosocial scores were chosen because of their summary nature, while the general fatigue scores were chosen as fatigue is increasingly being identified as an important symptom in pediatric cancer patients.^11^, ^12^, ^13^ We did not evaluate the PedsQL Acute Cancer Module in regression analysis because the numerous scales (namely pain and hurt, nausea, procedural anxiety, treatment anxiety, worry, cognitive problems, perceived physical appearance, and communication) would result in a multiple testing issue. Multiple testing can be problematic as it increases the likelihood of identifying statistically significant predictor variables by chance. We did not include the PedsQL Acute Cancer Module summary score as it is less clinically relevant since it is the compilation of the individual scales.

Potential predictors of QoL considered were age, gender, ethnicity, race, insurance status, risk status, random assignment to bortezomib, duration of neutropenia, and the total number of nonhematological grade 3‐4 Common Terminology Criteria for Adverse Event (CTCAE) submissions. The Children's Oncology Group requires adverse events to be submitted proximal to the completion of each chemotherapy cycle. All adverse events were clinician reported; none of the toxicities were self‐reported by patients or proxy‐reported by guardians as a patient‐reported CTCAE is not yet validated in children. CTCAEs were submitted by institutional clinical research associates and local investigators and only nonhematologic toxicities were collected for this trial. Submission was electronic. Two authors (RA and LS) monitored all submitted toxicities as they were submitted to optimize reporting quality. All Children's Oncology Group institutions are the subject to regular auditing visits in which data quality (including CTCAE submissions) are evaluated. CTCAE submissions were counted from starting each cycle of chemotherapy until the day the QoL assessment was obtained.

In order to evaluate concordance between proxy‐report and self‐report scores for the subset of patients with both self‐report and proxy‐report scores, we calculated the intraclass correlation coefficient (ICC) and its 95% confidence interval for each measure at each time point. This was calculated using a one‐way random effects model and reporting ICC(1,1) for each analysis.^14^ Concordance was defined as follow: ICC < 0.4—poor; 0.40‐0.59—fair; 0.60‐0.74—good; and 0.75‐1.00—excellent.^15^ The SAS statistical program (SAS for Windows, version 9.4; SAS Institute Inc, Cary, NC) was used to perform analyses. Statistical tests were 2‐sided, and a P‐value of less than 0.05 was considered statistically significant.

## 3 RESULTS

**Figure 1:**
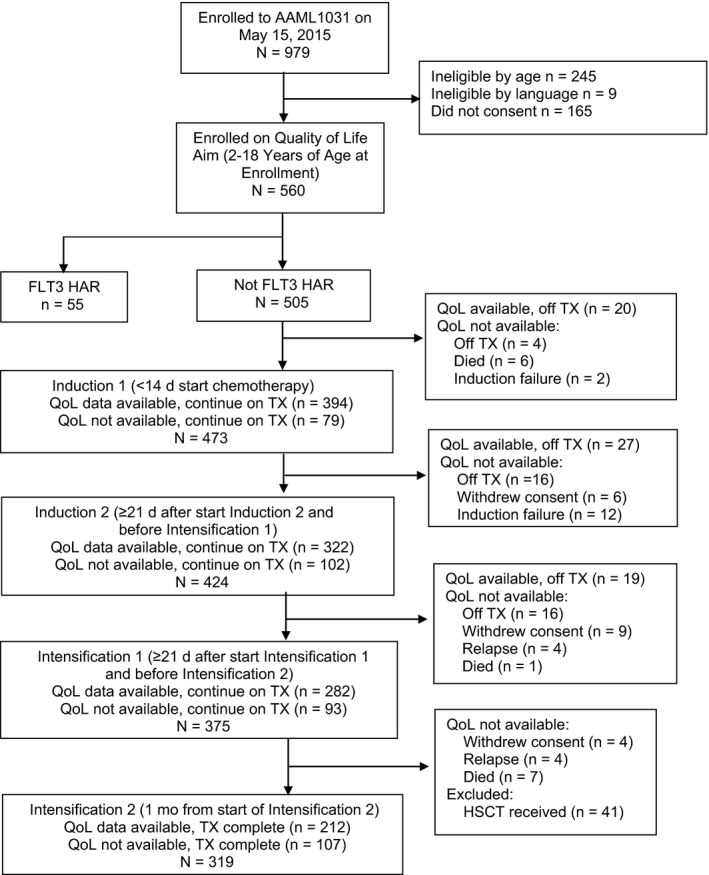
Flow chart of enrollment and attrition to the quality of life aim of AAML1031 among guardian proxy‐reports only. FLT3 HAR, FLT3 high allelic ratio; HSCT, hematopoietic stem cell transplantation; QoL, quality of life; TX, AAML1031 protocol treatment. *QoL data available means that at least one of the 3 PedsQL modules were submitted by the guardian

**Figure A1:**
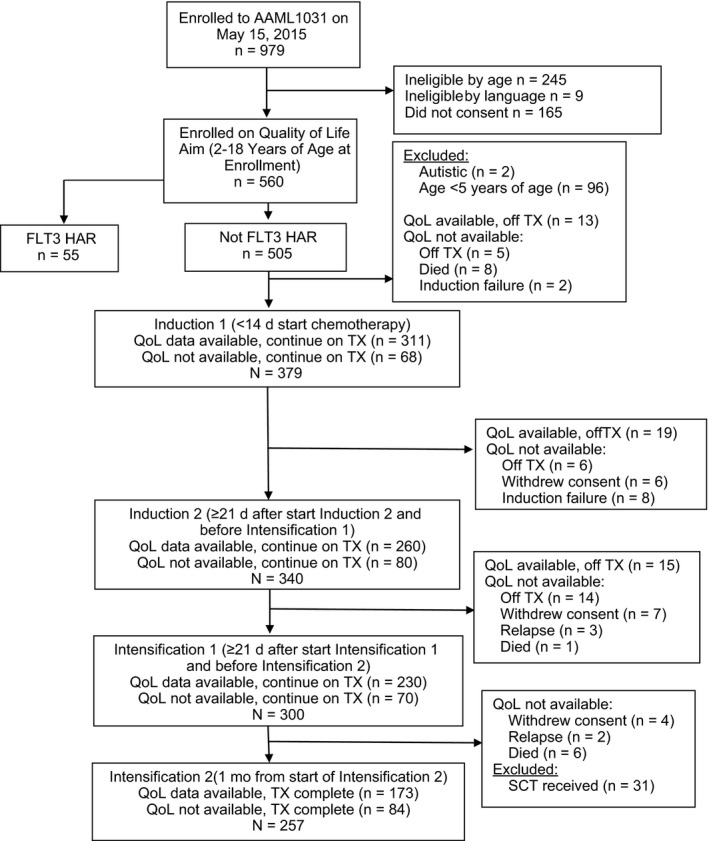
Flow chart of enrollment and attrition to the quality of life aim of AAML1031 among child self‐reports only. FLT3 HAR, FLT3 high allelic ratio; HSCT, hematopoietic stem cell transplantation; QoL, quality of life; TX, AAML1031 protocol treatment. *QoL data available means that at least one of the 3 PedsQL modules were submitted by the child

Figure 1 and Figure A1 illustrate the flow diagrams for guardian proxy‐report and child self‐report participation to, and attrition from the QoL aim. During the time frame that AAML1031 was accruing participants to the QoL aim (up until May 15, 2015), 979 patients were enrolled among which 725 were eligible for the QoL aim based upon age and language. There were 165 guardians who declined to participate and thus, 560 (77.2% participation) agreed to submit QoL proxy assessments. Among the 560 children of these guardians, 55 were FLT3 ITD HAR, thus leaving 505 guardian proxy‐report participants included in this analysis. From these guardians, 348 children agreed to submit self‐report assessments and provided at least one assessment.

Table 1 shows the demographics of the overall cohort of guardian and child respondents. Characteristics of children providing self‐report assessments were similar to the children of guardians providing proxy‐report assessments except for age in which those self‐reporting QoL were older.

**Table 1:** Demographics of study cohort for proxy and self‐report respondents

	Parent proxy‐report (N = 505)	Child Self‐Report (N = 348)
Characteristic		
Male gender	264 (52.3%)	185 (53.2%)
Age in years		
2‐4	99 (19.6%)	NA
5‐7	58 (11.5%)	50 (14.4%)
8‐12	127 (25.1%)	113 (32.5%)
13‐18	221 (43.8%)	185 (53.2%)
Race		
Asian	27 (5.3%)	16 (4.6%)
Black	60 (11.9%)	39 (11.2%)
White	362 (71.7%)	255 (73.3%)
Other	4 (0.8%)	3 (0.9%)
Unknown	52 (10.3%)	35 (10.1%)
Hispanic or Latino		
Yes	62 (12.3%)	37 (10.6%)
No	429 (85.0%)	300 (86.2%)
Unknown	14 (2.8%)	11 (3.2%)
Country		
United States	443 (87.7%)	312 (89.7%)
Canada	32 (6.3%)	14 (4.0%)
Australia/New Zealand	30 (5.9%)	22 (6.3%)
Insurance Status		
Private insurance	261 (51.7%)	190 (54.6%)
Medicaid	129 (25.5%)	86 (24.7%)
Medicare	14 (2.8%)	10 (2.9%)
Self pay or no insurance	10 (2.0%)	6 (1.7%)
Military sponsored	10 (2.0%)	6 (1.7%)
Other	64 (12.7%)	37 (10.6%)
Unknown	17 (3.4%)	13 (3.7%)
Risk Status		
Low risk	381 (75.4%)	272 (78.2%)
High risk	114 (22.6%)	74 (21.3%)
Unknown	10 (2.0%)	2 (0.6%)
Treatment assignment		
No bortezomib	267 (52.9%)	187 (53.7%)
Bortezomib	238 (47.1%)	161 (46.3%)

Abbreviations: FAB, French‐American‐British; NOS, not otherwise sub‐classified.

There were a total of 3757 guardian proxy‐report QoL module submissions. Table 2 shows the median QoL scores for the generic, cancer‐specific and fatigue measures, both for total scale scores and by specific scales as assessed by guardians. Across all time points, median generic total scale scores ranged from 60 to 70 while median cancer‐specific total scale scores ranged from 66 to 70. Conversely, median general fatigue scores were 50‐58. No clear trends over time were observed for any of these scores.

**Table 2:** Summary of quality of life scores over time for guardian proxy respondents showing median and interquartile ranges by time period^a^

	Induction 1: <14 d start chemotherapy	Induction 2: ≥21 d after start Induction 2 and before Intensification 1	Intensification 1: ≥21 d after start Intensification 1 and before Intensification 2	Intensification 2: 1 mo from start of Intensification 2
Number completing at least one assessment	414	349	301	212
Generic quality of life				
Total scale score	69.6 (54.2‐82.6)	64.7 (50.0‐77.3)	63.0 (47.8‐79.8)	60.0 (46.7‐77.2)
Physical health summary	65.6 (37.5‐84.4)	59.4 (37.5‐78.1)	56.3 (34.4‐78.1)	53.1 (34.4‐75.0)
Psychosocial health summary	73.3 (60.0‐85.0)	68.3 (55.0‐80.0)	67.3 (52.3‐81.7)	63.3 (51.7‐80.8)
Emotional functioning	65.0 (50.0‐80.0)	60.0 (50.0‐75.0)	60.0 (50.0‐75.0)	60.0 (50.0‐75.0)
Social functioning	90.0 (70.0‐100.0)	80.0 (65.0‐95.0)	80.0 (65.0‐95.0)	75.0 (60.0‐95.0)
School functioning	70.0 (50.0‐90.0)	55.0 (35.0‐80.0)	55.0 (35.0‐80.0)	55.0 (35.0‐80.0)
Cancer specific scores				
Total scale score	67.3 (53.7‐78.7)	70.0 (57.4‐80.6)	68.5 (54.6‐81.2)	66.3 (56.5‐80.6)
Pain and hurt	62.5 (37.5‐75.0)	62.5 (50.0‐87.5)	62.5 (50.0‐75.0)	62.5 (50.0‐75.0)
Nausea	60.0 (40.0‐80.0)	55.0 (40.0‐75.0)	55.0 (35.0‐70.0)	50.0 (35.0‐75.0)
Procedural anxiety	50.0 (16.7‐75.0)	66.7 (41.7‐91.7)	75.0 (33.3‐100.0)	66.7 (33.3‐91.7)
Treatment anxiety	75.0 (50.0‐100.0)	75.0 (58.3‐100.0)	75.0 (58.3‐100.0)	75.0 (58.3‐100.0)
Worry	75.0 (50.0‐100.0)	75.0 (50.0‐100.0)	75.0 (50.0‐91.7)	66.7 (50.0‐91.7)
Cognitive problems	75.0 (60.0‐95.0)	75.0 (58.3‐91.7)	75.0 (55.0‐93.8)	75.0 (55.0‐90.0)
Physical appearance problems	83.3 (58.3‐100.0)	83.3 (58.3‐100.0)	75.0 (58.3‐100.0)	83.3 (58.3‐100.0)
Communication problems	75.0 (50.0‐100.0)	75.0 (58.3‐100.0)	750 (58.3‐100.0)	75.0 (58.3‐100.0)
Fatigue				
Total scale score	59.7 (45.8‐73.6)	64.7 (50.0‐77.3)	62.5 (47.2‐77.8)	58.3 (44.4‐75.0)
General fatigue	50.0 (33.3‐70.8)	58.3 (45.8‐75.0)	56.3 (41.7‐75.0)	54.2 (33.3‐70.8)
Sleep rest fatigue	50.0 (33.3‐70.8)	58.3 (41.7‐75.0)	58.3 (37.5‐75.0)	54.2 (33.3‐75.0)
Cognitive fatigue	75.0 (58.3‐95.8)	75.0 (58.3‐95.8)	75.0 (54.2‐95.8)	70.8 (54.2‐95.8)

a Restricted to noninternal tandem duplication high‐allelic ratio patients only.

There were a total of 3009 self‐report QoL submissions. Table 3 shows the median QoL scores for the generic, cancer‐specific and fatigue measures, both for total scale scores and by specific scales as assessed by children themselves. Across all time points, median generic and cancer‐specific total scale scores were approximately 70 while general fatigue scores were 58‐67.

**Table 3:** Summary of quality of life scores over time for self‐reporting respondents showing median and interquartile ranges by time period

	Induction 1: <14 d start chemotherapy	Induction 2: ≥21 d after start Induction 2 and before Intensification 1	Intensification 1: ≥21 d after start Intensification 1 and before Intensification 2	Intensification 2: 1 mo from start of Intensification 2
Number completing at least one assessment	324	279	245	173
Generic quality of life				
Total scale score	68.9 (56.5‐80.4)	69.6 (56.5‐82.1)	69.0 (55.4‐83.7)	69.6 (54.3‐84.8)
Physical health summary	65.6 (43.8‐84.4)	65.6 (50.0‐84.4)	64.1 (43.8‐84.4)	62.5 (43.8‐84.4)
Psychosocial health summary	71.7 (61.7‐83.3)	71.7 (60.0‐83.3)	73.3 (60.0‐85.0)	73.3 (60.0‐86.7)
Emotional functioning	70.0 (55.0‐80.0)	70.0 (60.0‐88.8)	70.0 (55.0‐90.0)	75.0 (60.0‐90.0)
Social functioning	88.8 (70.0‐100.0)	85.0 (70.0‐100.0)	84.2 (70.0‐100.0)	85.0 (65.0‐100.0)
School functioning	65.0 (50.0‐80.0)	60.0 (45.0‐80.0)	60.0 (40.0‐80.0)	65.0 (50.0‐81.3)
Cancer specific scores				
Total scale score	69.2 (57.4‐79.6)	72.2 (60.2‐83.0)	72.2 (59.6‐86.1)	73.1 (60.2‐85.2)
Pain and hurt	75.0 (50.0‐87.5)	75.0 (50.0‐87.5)	75.0 (50.0‐100.0)	75.0 (50.0‐100.0)
Nausea	60.0 (50.0‐80.0)	60.0 (45.0‐80.0)	60.0 (50.0‐80.0)	65.0 (50.0‐85.0)
Procedural anxiety	58.3 (25.0‐83.3)	66.7 (33.3‐91.7)	66.7 (33.3‐91.7)	66.7 (33.3‐100.0)
Treatment anxiety	75.0 (58.3‐100.0)	83.3 (66.7‐100.0)	91.7 (66.7‐100.0)	91.7 (66.7‐100.0)
Worry	66.7 (50.0‐83.3)	66.7 (50.0‐91.7)	75.0 (50.0‐91.7)	75.0 (50.0‐91.7)
Cognitive problems	75.0 (55.0‐90.0)	75.0 (60.0‐90.0)	75.0 (60.0‐95.0)	75.0 (55.0‐95.0)
Physical appearance problems	83.3 (58.3‐100.0)	83.3 (66.7‐100.0)	83.3 (66.7‐100.0)	83.3 (66.7‐100.0)
Communication problems	83.3 (58.3‐100.0)	83.3 (66.7‐100.0)	83.3 (66.7‐100.0)	83.3 (66.7‐100.0)
Fatigue				
Total scale score	61.1 (48.6‐73.6)	63.9 (54.2‐80.6)	63.9 (50.0‐79.2)	63.9 (50.0‐81.3)
General fatigue	58.3 (45.8‐75.0)	66.7 (50.0‐83.3)	66.7 (50.0‐83.3)	66.7 (50.0‐79.2)
Sleep rest fatigue	50.0 (33.3‐66.7)	58.3 (41.7‐75.0)	54.2 (41.7‐75.0)	54.2 (41.7‐75.0)
Cognitive fatigue	75.0 (58.3‐91.7)	75.0 (58.3‐95.8)	75.0 (50.0‐95.8)	75.0 (54.2‐95.8)

There were 2561 nonhematological grade 3‐4 CTCAE toxicities reported. Table 4 illustrates the relationship between potential predictors and QoL via *β* ± standard error in multiple linear regression. The total number of CTCAE toxicities was significantly associated with worse physical health summary scores (−3.00 ± 0.69; *P* < 0.001) and general fatigue (−2.50 ± 0.66; *P* < 0.001). Older age was significantly associated with more general fatigue (−0.58 ± 0.25; *P* = 0.022). Gender, white race, Hispanic ethnicity, private insurance status, risk status, bortezomib assignment, and duration of neutropenia were not significantly associated with physical health summary score, psychosocial health summary score or general fatigue.

**Table 4:** Factors associated with worse physical health, psychosocial health, and general fatigue by proxy report

Variable	Physical summary score		Psychosocial summary score		General fatigue score	
	*β* ± SE^a^	*P* value	*β* ± SE^a^	*P* value	*β* ± SE^a^	*P* value
Male sex	1.03 ± 2.72	0.705	−0.72 ± 1.72	0.676	4.70 ± 2.59	0.070
Age in years	−0.40 ± 0.27	0.131	−0.28 ± 0.17	0.092	−0.58 ± 0.25	0.022
High risk	0.54 ± 3.23	0.868	1.91 ± 2.03	0.348	−0.87 ± 3.11	0.780
Bortezomib assignment	−2.15 ± 2.73	0.430	0.14 ± 1.72	0.934	−2.79 ± 2.61	0.285
Days of neutropenia	−0.08 ± 0.19	0.661	0.005 ± 0.11	0.967	0.07 ± 0.19	0.702
Number of submitted CTCAE toxicities	−3.00 ± 0.69	<0.001	−0.81 ± 0.41	0.050	−2.50 ± 0.66	<0.001
Insurance status private	2.85 ± 2.74	0.298	2.98 ± 1.74	0.087	−1.08 ± 2.64	0.684
White race	−1.23 ± 3.71	0.740	−1.19 ± 2.34	0.611	−0.43 ± 3.52	0.903
Hispanic ethnicity	−2.12 ± 4.41	0.631	−1.31 ± 2.77	0.635	3.77 ± 4.24	0.375

Abbreviations: CTCAE, Common Terminology Criteria for Adverse Events; SE, standard error.

a Conducted using repeated measures linear regression. All models include variable, time point and interaction of variable and time point

Table 5 shows the ICCs between proxy and self‐report scores across all measures. In general, they ranged from 0.4 to 0.6 depending on specific scale and time point but overall, the scores were consistent across QoL modules and scales.

**Table 5:** Concordance between parent proxy and child self‐report for children with acute myeloid leukemia

N=Number having both proxy and self‐reports scores available	Induction 1		Induction 2		Intensification 1		Intensification 2	
	N	ICC (95% CI)	N	ICC (95% CI)	N	ICC (95% CI)	N	ICC (95% CI)
Generic quality of life								
Total scale score	329	0.604 (0.531‐0.668)	285	0.523 (0.433‐0.602)	241	0.582 (0.492‐0.659)	159	0.594 (0.484‐0.686)
Physical health summary	329	0.506 (0.421‐0.582)	284	0.496 (0.403‐0.579)	240	0.616 (0.531‐0.688)	159	0.601 (0.492‐0.692)
Psychosocial health summary	329	0.593 (0.518‐0.659)	285	0.497 (0.404‐0.579)	239	0.517 (0.417‐0.604)	159	0.532 (0.411‐0.635)
Emotional functioning	327	0.480 (0.392‐0.559)	285	0.487 (0.394‐0.571)	238	0.503 (0.402‐0.592)	159	0.455 (0.222‐0.665)
Social functioning	328	0.499 (0.414‐0.576)	276	0.435 (0.334‐0.526)	235	0.435 (0.326‐0.533)	154	0.558 (0.439‐0.658)
School functioning	312	0.562 (0.481‐0.634)	240	0.510 (0.410‐0.597)	205	0.518 (0.410‐0.611)	139	0.521 (0.389‐0.632)
Cancer specific scores								
Total scale score	333	0.598 (0.525‐0.663)	285	0.655 (0.583‐0.717)	241	0.659 (0.582‐0.725)	161	0.619 (0.514‐0.706)
Pain and hurt	333	0.380 (0.284‐0.468)	284	0.599 (0.519‐0.668)	241	0.506 (0.405‐0.594)	161	0.565 (0.450‐0.661)
Nausea	331	0.440 (0.349‐0.523)	285	0.584 (0.502‐0.656)	241	0.470 (0.366‐0.563)	161	0.451 (0.319‐0.566)
Procedural anxiety	332	0.663 (0.598‐0.719)	283	0.693 (0.628‐0.749)	240	0.733 (0.668‐0.786)	158	0.656 (0.558‐0.737)
Treatment anxiety	331	0.501 (0.416‐0.578)	282	0.445 (0.346‐0.534)	237	0.570 (0.478‐0.650)	157	0.501 (0.374‐0.609)
Worry	329	0.472 (0.383‐0.552)	285	0.512 (0.421‐0.593)	240	0.501 (0.400‐0.590)	160	0.507 (0.382‐0.613)
Cognitive problems	332	0.497 (0.412‐0.574)	285	0.551 (0.465‐0.627)	239	0.610 (0.524‐0.684)	160	0.604 (0.496‐0.694)
Physical appearance Problems	321	0.497 (0.410‐0.575)	283	0.518 (0.427‐0.598)	236	0.482 (0.378‐0.574)	157	0.555 (0.437‐0.654)
Communication problems	326	0.473 (0.385‐0.553)	283	0.547 (0.460‐0.624)	236	0.484 (0.381‐0.576)	158	0.409 (0.271‐0.531)
Fatigue								
Total scale score	329	0.493 (0.407‐0.571)	282	0.590 (0.508‐0.661)	236	0.625 (0.540‐0.697)	160	0.581 (0.469‐0.675)
General fatigue	328	0.416 (0.322‐0.502)	282	0.488 (0.394‐0.572)	236	0.522 (0.423‐0.609)	159	0.499 (0.373‐0.607)
Sleep rest fatigue	329	0.475 (0.387‐0.554)	282	0.647 (0.574‐0.710)	236	0.590 (0.500‐0.667)	160	0.621 (0.516‐0.708)
Cognitive fatigue	329	0.486 (0.399‐0.564)	282	0.528 (0.438‐0.607)	235	0.561 (0.467‐0.642)	159	0.465 (0.334‐0.579)

Abbreviation: ICC, intraclass correlation coefficient.

## 4 DISCUSSION

In this large study including homogeneously and intensively treated pediatric cancer patients, we have described different aspects of QoL using both guardian proxy‐report and child self‐report approaches. The number of CTCAE toxicities was the primary factor influencing QoL among children with AML. Conversely, gender, white race, Hispanic ethnicity, private insurance status, risk status, bortezomib assignment, and duration of neutropenia were not significantly associated with physical health, psychosocial health or fatigue. This work is important as in general, descriptions of QoL to date in pediatric AML have focused on survivors^16^, ^17^, ^18^ rather than those receiving active treatment.

In this study, median proxy‐report generic total scale scores ranged from 60 to 70 while median cancer‐specific total scale scores ranged from 66 to 70. When comparing these values to proxy‐report in a general oncology sample across all diagnostic groups with no comorbid disease, mean scores were very similar for generic total scale score, which was 69.7 and cancer‐specific scores, which ranged from 60 to 78 for individual scales.^9^ This finding suggests that overall, in spite of children with AML requiring very intensive chemotherapy with associated prolonged and profound neutropenia and frequent hospitalizations, their generic and cancer‐specific QoL is not markedly different compared to a general oncology sample.

Conversely, median proxy‐report general fatigue scores were 50‐58 and median self‐report general fatigue scores were 58‐67. These are both lower compared to a general oncology sample in which mean proxy‐report general fatigue was 74 and self‐report general fatigue was 75.^9^ This finding suggests that the one domain that might be particularly affected in pediatric AML is fatigue. Consequently, identifying effective interventions to reduce fatigue should be a priority for pediatric AML chemotherapy recipients.^12^

We also found that the most important factor associated with worse physical health and fatigue was the number of CTCAE submissions. This suggests that if we can identify approaches to reduce these toxicities, we might be able to improve QoL in this population. There are evidence‐based guidelines to reduce toxicities and these are available at https://www.childrensoncologygroup.org/index.php/cog-supportive-care-guidelines. Evaluating compliance with guidelines is an important future initiative.

We also found that older age was associated with more fatigue. This finding is consistent with others.^11^ Interestingly, we did not find that QoL was associated with AML risk status or duration of neutropenia. In AAML1031, high‐risk patients received mitoxantrone and cytarabine as their second cycle rather than cytarabine, daunorubicin and etoposide, with the former being considered more intensive. This finding suggests that neutropenia in itself and presumably an increased infectious risk did not translate to differences in QoL. We also did not find that race, ethnicity and socioeconomic status as measured by insurance status significantly influenced QoL; these are encouraging findings in terms of equity of care.

Concordance in QoL between guardian proxy‐report and child self‐report was generally fair to good with ICC being >0.4‐0.6 in general. In a systematic review evaluating agreement between parents and children, correlation as measured using correlation coefficients or ICC was generally 0.3‐0.5 or >0.5 but was <0.3 in some studies.^19^ This may suggest that proxy‐report is a better surrogate for self‐report QoL in pediatric AML compared to other populations, although this does not negate the importance of obtaining self‐report if possible. A hypothesis could be given the intensive nature of chemotherapy and requirement for hospitalization, guardians of pediatric AML patients may spend more time with their children compared to healthier populations and thus, may be better raters of their child's health. To date, QoL assessment has focused on choosing a self‐report or proxy‐report approach with both having important limitations. Using a hybrid approach to QoL assessment, where guardians and children complete assessments together, could be a novel approach to resolving this dilemma and could be a focus of future work.

The major strength of our study was the large, homogeneously treated sample of children treated at multiple institutions, which improves the generalizability of our findings. In addition, we had the ability to test factors such as AML risk assignment and allocation to bortezomib, and their impact on QoL. However, our report must be interpreted in light of its limitations. The major limitation is that self‐report was optional and likely, those who agreed to self‐report were a biased sub‐population of the entire cohort. A second limitation is that this study was limited to English‐speaking families and different observations may occur in families who speak other languages. Third, a large number of proxy‐report and self‐report assessments were not submitted even though the participant remained eligible to submit QoL assessments. This speaks to the substantial issue of obtaining QoL assessments over a large number of institutions when children are frequently medically ill. Likely, it was the sickest children for whom reports were not submitted and thus, our QoL estimates may be too high. However, this also stresses that fatigue may actually be worse than what we reported given the likely direction of bias. Finally, the recall period was 4 weeks in this study. This may introduce recall bias and likely only reflects health more proximal to instrument completion. Using a 1 week recall version may have been a better approach to QoL assessment.

In conclusion, the number of CTCAE toxicities was the primary factor influencing QoL among children being treated for AML. Fatigue appears to be particularly affected during AML chemotherapy. Reducing toxicities should improve QoL and identifying approaches to ameliorate them should be a priority.

## DATA AVAILABILITY STATEMENT

The data that support the findings of this study are available from the corresponding author upon reasonable request.
